# Supplementary material for: The group-housed pigs attacking and daily behaviors detection and tracking based on improved YOLOv5s and DeepSORT
Source: PLoS One. 2025 Oct 31;20(10):e0334783. doi: 10.1371/journal.pone.0334783 (PMC12578152; doi:10.1371/journal.pone.0334783)
Supplement: Attachment — Submitted filename: renamed_1b8c5.docx [file pone.0334783.s001.docx]

5. Review Comments to the Author

Please use the space provided to explain your answers to the questions above. You may also include additional comments for the author, including concerns about dual publication, research ethics, or publication ethics. (Please upload your review as an attachment if it exceeds 20,000 characters)

Reviewer #1: This paper proposes a method based on improved YOLOv5s and DeepSORT for attacking and daily behaviors detection and tracking of group-housed pig to enhance livestock production efficiency and animal welfare. Overall, the research content of this paper is practical. However, the effectiveness of the proposed method still requires more rigorous experimentation for validation. Additionally, the theoretical analysis in the experimental section needs to be further deepened and expanded to strengthen the scientific and persuasiveness of the study. Therefore, it is suggested that the author revise and improve the article before submitting it for review again.

1、In Figure 4, there are two “IOU Match” processes performed. However, the second matching process is not described in the text. Please carefully review the figure and text to ensure consistency.

**Response:** Thank you for your careful review and insightful comment. We agree with your observation. In response, we have revised the manuscript to explicitly describe the second round of IOU matching shown in Figure 4. Specifically, in Section 2.2.2 “The improved DeepSORT tracker”, we added the following sentence:

“To further improve the matching accuracy, a second round of IOU matching is performed between the remaining unmatched tracks and unmatched detections. This step helps reduce ID switching caused by short-term occlusion or fast movements. The successfully matched tracks are also updated with KF.”

In addition, we revised the following part to reflect this update more clearly:

“(3) For unmatched detections that remain after the two rounds of IOU matching, ...”

These revisions ensure consistency between the text and Figure 4 and clarify the purpose and function of the second IOU matching step in our improved DeepSORT process.

2、In Figure 4, please clarify what parameter “age” refers to in the context. Additionally, the drawing logic of Figure 4 is not clear enough. It is suggested that the author redraw this figure.

**Response：**Thank you for your thorough review and valuable comments on our paper. In response to this, we have revised the manuscript to explicitly describe the meaning of "age" and provide additional context. Specifically, in Section 2.2.2 "The Improved DeepSORT Tracker," we added the following content:

*"For unmatched tracks, we deleted them when the age of tracks exceeds the max age (set as a maximum of 30 frames in this paper)." The term 'age' refers to the number of frames since the track was last matched with a detection.*

These revisions ensure consistency between the text and Figure 4 and provide a clearer explanation to help better understand the "age" parameter and the logic behind track management.

3、In the section 2.2.2, the author aims to reduce the generation of new tracks by evaluating the relationship between “n” and “tracks”, where “n” is determined by the average of the number of detected objects in nearest 3 frames. However, the duration of three frames in a video is very short, possibly less than 0.2 seconds. In practical environments, the loss of tracking due to occlusion can last for several seconds or even minutes. Therefore, when occlusion exceeds three frames, “n” may be smaller than the actual number of pigs. When occluded pigs reappear, it remains unclear whether the proposed method can still achieve effective tracking. The author is recommended to include additional experiments or theoretical analyses at an appropriate location to validate the effectiveness of the method under occlusion conditions that exceed three frames.

**Response：Thank you for your suggestion. In our experiments, using consecutive 3 frames of the video just confirm the trajectory from** **detected objects, and its effect is not very significant for the tracking results when we take the values 4 or 5 under normal circumstances or occlusion. *In addition, there will be a matching track (n) smaller than the actual number of pigs under*** *occlusion****, we will not delete the trajectories immediately at this time, we will preserve these tracks whether the occluded objects appear or not. We delete the track after 30 frames of matching failure.* Considering the reviewer's suggestion that ‘tracking loss due to occlusion may last for several seconds or even minutes’, we later adopt deleting the track after 300-500 frames of failed matching under occlusion conditions.**

4、The author demonstrates the effectiveness of the method in nighttime scenarios in Figure 6. The primary impact of nighttime conditions on model performance lies in the blurring of object features caused by low light, which subsequently affects the recognition and tracking capabilities of model. However, in the experimental scenarios of Figure 6, the lighting conditions are relatively adequate and the object features are obvious. Therefore, the validity of using these scenarios to verify the model performance under low-light conditions is questionable. It is recommended that the author redesign this part of the experimental content to effectively validate the model performance in low-light environments.

**Response：**Thank you for your valuable feedback. Based on your comments, we have further optimized and clarified the experiment. *“In Figure 6, YOLOv5s shows false negatives and false positives when the pigs are clustered together, whereas our improved method effectively addresses these issues by incorporating the C3CBAM attention module.*

*Specifically, in the first row of the experimental results, YOLOv5s fails to recognize the attack behavior due to missed detection when pigs are clustered together, while our improved method successfully detects the behavior (as indicated by the yellow arrow). In the second row, YOLOv5s incorrectly detects a standing pig as lying down, while our method correctly detects the standing pig (as shown by the orange arrow). These improvements are attributed to the application of the C3CBAM attention mechanism, which enhances the model's feature extraction ability in complex scenarios, improving detection accuracy.”*

We believe these improvements can effectively enhance object detection performance in clustered situations, especially in pig behavior analysis. Once again, thank you for your feedback. We will continue to optimize and improve our research.

5、In Figure 6, there are obvious errors in behavior recognition. For example, the “stand” is misidentified as “lie”. Moreover, the errors indicated by the yellow and orange arrows in the figure are clearly not caused by occlusion, which contradicts the analysis provided in the text. It is recommended that the author carefully verify the experimental results and conduct a correct theoretical analysis based on them to enhance the credibility of the article.

**Response：**Thank you for your valuable feedback. Regarding the behavior recognition errors mentioned in Figure 6, we have conducted a thorough review and made revisions. *We have ensured that the descriptions in the figure are consistent with the experimental results, particularly addressing the issue of misidentifying a "standing" pig as "lying down." We have reanalyzed the relevant experiments and adjusted the description to make it more accurate. Additionally, we have corrected the analysis in the text to align with the experimental results, ensuring that the theoretical analysis is consistent with the findings, thereby enhancing the credibility of the article. Thank you again for your comments.* We will continue to improve our work to ensure the accuracy and scientific validity of the content.

6、Figure 7 demonstrates the effectiveness of the model in crowded scenarios. However, the figure does not depict a high-density environment. In fact, the stocking density is even lower than that shown in Figure 6. Therefore, the effectiveness of the model in high-density environments remains to be verified. It is recommended that the author reselect test images and revise the corresponding experimental and analytical content.

**Response：**Thank you for your valuable feedback. In response to your comment regarding the stocking density in Figure 7, we have revised and updated the experimental results. We acknowledge that Figure 7 does not depict a high-density environment but rather a scenario with a relatively larger number of pigs. Based on this, we have adjusted the description to more accurately reflect the experimental results. Specifically, in the description of Figure 7a, we have made a clearer distinction between scenes with fewer and more pigs to ensure a more accurate correspondence between the figure and the text. Thank you for your careful review, and we will continue to refine the experimental description to ensure consistency and accuracy between the figure and the text.

we revised the following part as follows:

*“Fig. 7 illustrates several detection results of the improved YOLOv5s on the test set in different scenarios. From the results in Fig. 7b, it can be seen that with the use of the C3CBAM attention mechanism, our method effectively overcomes false detection and missed detection issues. In the first row of Fig. 7a, as indicated by the orange arrow, the YOLOv5s detector causes false detection when the number of pigs is low. In the second row of Fig. 7a, as indicated by the yellow arrow, the YOLOv5s detector suffers from missed detection when the number of pigs is high. In contrast, the improved method maintains high detection accuracy across different scenarios, whether there are fewer or more pigs, and effectively avoids both false and missed detections. Therefore, the improved method, based on the C3CBAM attention mechanism, provides more stable and precise detection results in complex scenarios, further enhancing the robustness and practicality of the model in dynamic environments.”*

(a) YOLOv5s	(b) improved YOLOv5s

Fig.7 Example detection results between YOLOv5s and improved YOLOv5s under different scenes.

7、In this study, the author has achieved the task of behavior classification of group-housed pigs through object detection. However, object detection only considers the spatial information of behaviors and lacks temporal information. The author also states in Table 1 that aggressive behavior is a dynamic process. So, how does the proposed method achieve accurate recognition without temporal information? For example, in Figure 7, two pigs with their heads close together were identified as standing. Similarly, another two pigs were identified as aggressive behavior. Why can the proposed method distinguish behaviors through the spatial relationships in the single frame image. Therefore, it is suggested that the author add a section in the introduction or experimental part to explain how the study ensures the accuracy of behavior detection in the absence of temporal information.

**Response：Thank you for your valuable comments. Regarding the issue of achieving behavior classification without temporal information, we have adopted the** **following strategies in our study to ensure classification accuracy:**

***(1) Utilization of Spatial Relationships: Although our model performs detection based on single-frame images, the improved YOLOv5s and C3CBAM attention mechanism effectively capture the relative spatial relationships between pigs. For example, when two pigs' heads are close together, the model can identify the behavior as standing rather than aggressive.***

***(2) Improved Feature Extraction: By incorporating the C3CBAM attention module, the model not only focuses on local spatial information but also effectively extracts more discriminative behavioral features based on the relative relationships between pigs, thus reducing misclassifications.***

***(3) Dynamic Behavior Modeling: While we do not explicitly use temporal information, we simulate certain features of dynamic processes by statistically analyzing the position changes of pigs across consecutive frames. This helps the model better understand the continuity and changes in behavior, especially in group behaviors.***

**Therefore, despite the absence of temporal information, our method can accurately distinguish pig behaviors in single-frame images through spatial relationships and feature extraction techniques. Once again, thank you for your feedback. We will further refine the content based on your suggestions.**

Reviewer #2: Below are my comments about the paper:

1. The integration of CBAM and Shape-IoU into YOLOv5s, while effective, is not fundamentally novel. Both CBAM (a well-established attention mechanism) and Shape-IoU (a recent but generic loss function) have been applied in other domains. The authors do not sufficiently justify why these components are uniquely suited to pig behavior detection compared to alternative attention mechanisms (e.g., SE, ECA) or IoU variants (e.g., CIoU, DIoU). The novelty lies primarily in their application to this specific use case, but this is not strongly differentiated from prior agricultural CV studies (e.g., reference 21 uses rotated bounding boxes, which might offer better occlusion handling).

**Response：**Thank you for your detailed review and valuable comments on our paper. In response to your feedback, we have revised the manuscript and further clarified the reasons for integrating CBAM and Shape-IoU into YOLOv5s, particularly regarding their application in pig behavior detection. The relevant section 2.2.1 "Proposed Improved YOLOv5s Detector" has been updated, with the revised content as follows:

*"In order to address this issue, this study integrates the CBAM (Channel and Spatial Attention Module) and Shape-IoU loss function into YOLOv5s to enhance its performance in pig behavior detection. Although CBAM and Shape-IoU have been widely used in other domains (such as image classification and object detection), their application in pig behavior detection offers unique advantages. Compared to other attention mechanisms, CBAM effectively focuses on the key information in images, especially in farming environments where lighting variations and occlusions significantly impact detection accuracy. By adaptively adjusting channel and spatial attention, CBAM helps the model better understand target features in complex backgrounds, thus improving detection accuracy in complex scenarios.*

*Additionally, Shape-IoU, as a novel loss function, provides more precise optimization of the target bounding box shape compared to traditional IoU metrics (such as CIoU and DIoU). In pig farming scenarios, the posture and spatial variations of pigs are complex, and traditional IoU loss may not adequately handle these variations. Shape-IoU, by more accurately calculating the overlap of target shapes, effectively improves the regression accuracy of the detection box, especially when targets are occluded or partially overlapping.*

Therefore, by integrating CBAM and Shape-IoU into YOLOv5s, this study not only improves the model's detection accuracy in dense farming environments but also maintains high detection stability under complex conditions such as lighting changes and target occlusions."

These modifications ensure that the novelty and advantages of integrating CBAM and Shape-IoU into YOLOv5s are fully explained, clearly demonstrating their unique contribution to pig behavior detection.

2. While the improved YOLOv5s outperforms older YOLO versions (v8, v9, v10) in Table 6, this comparison lacks depth. For instance, ORP-Byte (reference 21) achieves MOTA of 99.8%, significantly higher than the 94.5% reported here. The authors should address why their method underperforms in tracking accuracy despite architectural improvements.

**Response 2：**Thank you for your valuable and in-depth feedback on our research. We acknowledge that although our improved YOLOv5s outperforms older versions of YOLO (v8, v9, v10) in Table 6, our model falls short in tracking accuracy compared to ORP-Byte (reference 21). We have conducted further analysis regarding this issue.

*Firstly, ORP-Byte and our method differ in terms of optimization goals and application scenarios. ORP-Byte achieves a very high MOTA in multiple object tracking (MOT) tasks, which is closely related to its dedicated optimizations for tracking performance. In contrast, our study focuses on pig behavior detection, especially under complex farming environments. In such settings, challenges such as lighting variations, occlusions, and high target density can significantly affect tracking performance.*

*Secondly, although architectural improvements were made—such as the integration of CBAM and Shape-IoU—our model, while achieving higher detection accuracy, still encounters challenges in tracking under complex scenarios. In future work, we plan to further optimize the tracking module, particularly by incorporating more advanced tracking algorithms and strategies to improve overall tracking accuracy.*

*In conclusion, although our model underperforms ORP-Byte in certain aspects, we believe our approach offers unique advantages in the specific context of pig behavior detection, especially within densely populated farming environments. We will continue refining our method to close this performance gap in future work.*

3. The ID-switch reduction mechanism in DeepSORT, which leverages the constant number of pigs per pen, is pragmatic but not groundbreaking. Similar constraints (e.g., fixed herd sizes) have been exploited in prior livestock tracking work (e.g., reference 20). The paper does not quantify how this modification compares to existing trackers (e.g., ByteTrack, FairMOT) in similar settings.

**Response：Thank you for your valuable comments.** We have compared the results with existing trackers (e.g., ByteTrack and OC-SORT) in similar settings, as shown in Table 7. FairMOT tracker does not have behavioral tracking, therefore, we used a more advanced OC-SORT tracker to complete the comparison experiments. The revised content is added in the discussion section.

The added comparison results of improved DeepSORT with other trackers are as follows:

***“Table 7 presents a comparison of tracking results for improved DeepSORT, ByteTrack, OC-SORT, and DeepSORT. It is observed that improved DeepSORT outperforms other tracking algorithms in all metrics. Compared to ByteTrack, OC-SORT, and DeepSORT, our method achieves improvements in MOTA by 4.6%, 3.4%, 9.4%, in IDF1 by 2.9%, 2.3%, 3.7%, and in MOTP by 7.3%, 2.6%, 8.2%, respectively. Furthermore, our method shows the best performance in IDs with only 216. Overall, our method achieves the best results in IDF1, IDs, MOTA, and MOTP, which demonstrates the superior identity consistency of our approach, leading to more stable and reliable tracking performance.”***

Table 7 The comparison results of improved DeepSORT with other trackers.

| trackers | IDF1↑ | IDs↓ | MOTA↑ | MOTP↑ |
| --- | --- | --- | --- | --- |
| ByteTrack | 69.9 | 278 | 89.9 | 87.6 |
| OC-SORT | 70.5 | 262 | 91.1 | 92.3 |
| DeepSORT | 69.1 | 299 | 85.9 | 86.7 |
| Improved DeepSORT | **72.8** | **216** | **94.5** | **94.9** |

4. The "attack" behavior has only 65 pairs pre-augmentation, raising concerns about model generalizability.

**Response：**Thank you for pointing out concerns regarding the number of samples for the "attack" behavior. Based on your suggestion, we have clarified the issue regarding the number of "attack" behavior samples and provided additional details on the data augmentation measures taken. *The "attack" behavior category in our study includes four subtypes: (1) two pigs with head contact and rapid head bobbing (65 pairs), (2) one pig with head contact to the body of another pig (85 pairs), (3) two pigs moving rapidly towards each other (575 pairs), and (4) one pig biting the tail of another pig (224 pairs). Although the first two subtypes have relatively fewer samples, the total number of "attack" behavior instances before data augmentation is 949 pairs, which provides a sufficient foundation for model training and validation.*

*To further enhance the model’s robustness, we applied various data augmentation techniques to the original "attack" behavior samples, thereby increasing their diversity and effective quantity. Additionally, a class-balanced loss strategy was employed during training to mitigate the impact of data imbalance. These measures together ensure that the model achieves good generalizability in recognizing and tracking aggressive behaviors among group-housed pigs.*

*These additional explanations help to better clarify the work done in handling "attack" behavior samples, ensuring that our approach effectively addresses the issue of sample imbalance and improves the model's generalization capability.*

5. The study emphasizes accuracy but omits computational efficiency metrics (e.g., FPS, GPU memory usage). For farm applications, real-time processing is critical.

**Response：**Thank you for your suggestion. We have added the computational efficiency metrics, as shown in Table 3. The added results of computational efficiency metrics are as follows:

*“The results of the five methods are similar in terms of computing speed FPS and GFLOPs metrics, and there is no significant difference in speed.”*

**Table 3**. Comparison of YOLOv5s with different strategy methods.

| CBAM | C3CBAM | Shape-IoU | Precision↑ | Recall↑ | mAP@0.5↑ | FPS | GFLOPs |
| --- | --- | --- | --- | --- | --- | --- | --- |
| − | − | − | 94.7 | 96.6 | 96.2 | 43.8 | 15.8 |
| √ | − | − | 95.7 | 97.1 | 97.3 | 41.1 | 16.1 |
| − | √ | − | 97.3 | 98.5 | 98.7 | 40.9 | 16.0 |
| √ | − | √ | 96.1 | 97.5 | 97.9 | 41.1 | 16.1 |
| − | √ | √ | **98.2** | **99.2** | **99.1** | 40.9 | 16.0 |

6. The paper does not mention key limitations, such as dependency on fixed camera angles, sensitivity to extreme occlusions, or scalability to larger herds.

**Response：**Thank you for your suggestion regarding the camera angle. The video surveillance system used in our data collection process (as shown in Fig. 1(a)) places the camera in the middle of the roof, ensuring broad coverage of the pig group's activities. We have further clarified this point in the revised manuscript to explain the choice of a fixed camera angle. Although we rely on a fixed camera angle, this setup has proven effective in capturing the pigs' behaviors in the context of this study.

*At the same time, we acknowledge that in larger herds or more complex environments, a fixed angle may present some potential limitations. Therefore, in the revised manuscript, we have added suggestions for future research, proposing the exploration of more flexible camera layouts or multi-camera setups to enhance the system's scalability and adaptability to complex scenarios.*

*These revisions help further clarify our consideration of this issue and provide guidance for future research directions.*
