# Supplementary material for: The group-housed pigs attacking and daily behaviors detection and tracking based on improved YOLOv5s and DeepSORT
Source: PLoS One. 2025 Oct 31;20(10):e0334783. doi: 10.1371/journal.pone.0334783 (PMC12578152; doi:10.1371/journal.pone.0334783)
Supplement: Attachment — Submitted filename: renamed_14002.docx [file pone.0334783.s002.docx]

Please use the space provided to explain your answers to the questions above. You may also include additional comments for the author, including concerns about dual publication, research ethics, or publication ethics. (Please upload your review as an attachment if it exceeds 20,000 characters)

Reviewer #1: The paper is improved from the previous version. However, it still has some issues that require further refinement.
(1) Although the authors have provided explanations regarding the second IOU-based association strategy in their response and the manuscript, the relevant descriptions remain insufficiently clear. Specifically, what are the technology or theoretical foundations that differentiate the first IOU-based association strategy from the second one proposed by the authors? Why is it that, by employing the IOU association method again for the unmatched trajectories and detection bounding boxes after the first association, a match can be achieved? The authors need to further expound why repeating the IOU matching process once more can effectively address the identity switching challenge caused by occlusion.

**Reponse:** Most multi-object tracking (MOT) methods obtain identities by associating detection boxes whose scores are higher than a threshold (between 0.5 and 0.8). The objects with low detection scores (<=0.4), e.g. occluded objects, are simply thrown away, which brings non-negligible true object missing and fragmented trajectories.

**The data association strategy proposed in our paper** **retains all detection boxes and performs identity (ID) matching**, dividing them into high-confidence and low-confidence groups. Firstly, the high-confidence detection boxes are associated into trajectories (the first association matching), and then the low-confidence detection boxes are associated with unmatched tracking objects to retain the low-confidence detection boxes and filter out the background (the second matching).

**The technology foundation of this strategy is based on reference [1,2]**. By leveraging the similarity between detection boxes and tracking trajectories, while retaining high-score detection results, background is removed from low-score detection results to unearth genuine objects (such as those that are occluded or blurred), thereby reducing missed detections and enhancing the continuity of trajectories[3-5].

**The following is a specific example to illustrate the application and process changes by employing the IOU association method again**.

Firstly, according to the Fig.1 (Examples of our method that associates every detection box) from reference [2], in frame t1, we initialize three different tracklets as their scores are all higher than 0.5. as shown in Row (a) of Fig. 1.

Then, **due to the complex scenarios in videos, detectors are prone to make imperfect predictions.** High-score detection boxes usually contain more true positives than low-score ones. However, simply eliminating all low-score boxes is sub-optimal since low-score detection boxes sometimes indicate the existence of objects, e.g. the occluded objects. Filtering out these objects causes irreversible errors for MOT and brings non-negligible missing detection and fragmented, as shown in Row (b) of Fig. 1.

Finally, we perform the second association between the unmatched tracklets and the low-score detection boxes using the same motion similarity to recover the true objects and remove the background. The association result is shown in line (c) of Fig. 1.

Fig. 1. Examples of our method that associates every detection box. (a) shows all the detection boxes with their scores. (b) shows the tracklets obtained by previous methods that associate detection boxes whose scores are higher than a threshold, i.e. 0.5. The same box color represents the same identity. (c) shows the tracklets obtained by our method. The dashed boxes represent the predicted box of the previous tracklets using Kalman filter. The two low-score detection boxes are correctly matched to the previous tracklets based on the large IoU. The number colored in yellow denotes the score of the box.trajectories

[1] Y. Zhang, P. Sun, Y. Jiang, D. Yu, F. Weng, Z. Yuan, P. Luo, W. Liu, and X. Wang, “Bytetrack: Multi-object tracking by associating every detection box,” in ECCV. Springer, 2022, pp. 1–21.

[2] Yifu Zhang, Xinggang Wang, Xiaoqing Ye, Wei Zhang, Jincheng Lu, Xiao Tan, Errui Ding, Peize Sun, Jingdong. WangByteTrackV2: 2D and 3D Multi-Object Tracking by Associating Every Detection Box. In Proceedings of ICCV, 2023 (arXiv:2303.15334v1).

[3] J. Pang, L. Qiu, X. Li, H. Chen, Q. Li, T. Darrell, and F. Yu.Quasi-dense similarity learning for multiple object tracking. In Proceedings of the IEEE/CVF Conference on ComputerVision and Pattern Recognition, pages 164–173, 2021.

[4] Y. Zhang, C. Wang, X. Wang, W.Zeng, andW.Liu. Fairmot: On the fairness of detection and re-identification in multiple object tracking. arXiv preprint arXiv:2004.01888, 2020.

[5] Z. Wang, L. Zheng, Y. Liu, Y. Li, and S. Wang. Towards real-time multi-object tracking. In Computer Vision–ECCV2020: 16th European Conference, Glasgow, UK, August 2328, 2020, Proceedings, Part XI 16, pages 107–122. Springer,2020.

(2) The authors employed a specially designed association strategy in the tracking module, effectively addressing the identity switching issue caused by occlusion. However, in the experimental section, we did not find relevant visualization results to verify the effectiveness of this improvement. Could the authors demonstrate the differences before and after the improvement through visualizations of consecutive frames or trajectory plots, so as to validate the effectiveness of reducing identity switching, rather than relying solely on single-frame images and metrics for illustration?

Reponse: **Thank you for your suggestion. We demonstrate the differences before and after the improvement through visualizations of consecutive frames to validate the effectiveness of reducing identity switching.**

The values of ID6 (shown in green arrows) are correct when 20 frames from the Pig09 video are not occluded using the unimproved and improved DeepSORT methods, as shown in the first line of Figure 10. However, at 113 frames, using the unimproved DeepSORT, the values of ID6 under occlusion mistakenly become 22 (shown in red arrows); and until 150 frames, the value of ID6 mistakenly switch to 22 (shown in red arrows). Using the improved DeepSORT method, no ID error switch has occurred, as shown in the second and third lines of Figure 10.

Fig.10 Comparison results between DeepSORT of improved DeepSORT model on occlusion conditions.
